# The Life of Pierre Fauchard (1678-1761)*Read at the Celebration of the Bicentenary anniversary of the publication of Fauchard’s work, held at the Sorbonne in Paris, December 16, 1922.

**Published:** 1923-08

**Authors:** George Viau

**Affiliations:** Paris, France, Professor and President of the Dental School of Paris


### DENTAL REGISTER

THE MAGAZINE OF DENTISTRY

Vol. LXXVII AUGUST, 1923 No. 8

### THE LIFE OF PIERRE FAUCHARD (1678-1761).*

BY GEORGE VIAU, D. E. D. P., PARIS, FRANCE

Professor and President of the Dental School of Paris

There are times, in the course of the centuries, when a man arises endowed with a superior intelligence, a great capacity for labor and, may I venture to say, a sort of prescience. As a result of the effort of such a mind the branch of human knowledge to which he has devoted himself achieves a progress which several generations would not suffice to consummate.

Such was Pierre Fauchard, who practiced our profession two hundred years ago.

While Fauchard did not originate all he unfolds in his remarkable book, at the same time we must admit that in utilizing the unassuming works of his predecessors he made use of them merely as "watch-towers whence to gaze afar," to quite the delightful and picturesque simile of Ambrose Paré! Not content with methodically arranging the scattered works in existence before his day and turning them into a homogeneous body of doctrines, his pioneer spirit adventured into other arts and trades to cull from them instruments and processes which he adapted to his professional needs. Thus he needs mechanics, watchmaking, jewelry, enameling, etc., to contribute to the development of dental prosthesis, which previously had been in a rudimentary state.

* Read at the Celebration of the Bicentenary anniversary of the publication of Fauchard's work, held at the Sorbonne in Paris, December 16, 1922.

In the realm of dental surgery and of operative dentistry, which joined to prosthesis give him his full professional value, Fauchard, besides noting and co-ordinating the knowledge already acquired, created new curative methods.

Nevertheless, while in this direction he proved exceedingly bold, he never departed from good sense and clear judgment.

He remained always and above all a man of observation and an exponent of the experimental system. Nothing in his work is obscure; all is minutely described and clearness is not the least of his merits so long as he does not approach the nebulous medical theories of his epoch. On reading his book one regrets that a man of his type did not have a wider field of activity and the means to instruct larger classes of students in the science which he described so well. He possessed in the highest degree the two qualities of the true teacher; practical good sense and lucidity of expression.

It may be said that with Fauchard dentistry entered into its true and almost final path. The considerable legacy left by this great ancestor, which has not ceased to grow to the present hour, renders the task of him who would exercise our profession creditably and usefully an arduous and complex one, for he must join to a long period of study a manual dexterity of the highest order. Thus only may he become a practitioner worthy of the name.

The life of Pierre Fauchard, like the lives of many other men of genuis, had been more or less surrounded by mystery, and it is the investigations meant to supply this lack which constitute the subject of the present address.

The birthplace of Pierre Fauchard was unknown, but we were aware that he was born in Brittany at a date which may be placed as between 1675 and 1680.

As a matter of fact, he states in his book "The Surgeon-Dentist or Treatise on the Teeth" that being in Angers in 1696 he performed a series of operations, very daring for that period, to regulate the jaws of one Crépy de la Mabilière. The operation consisted of the extraction of the four cuspid teeth and subsequent displacement of the incisors by means of the key to move them into normal position, and their retention by means of waxed thread. It is evident that to attempt an operation of this nature implied the possession of a steady hand combined with ripe professional experience. For these reasons at least we may conclude that Fauchard was not less than twenty years old in that year. Moreover his reputation was favorably known to the family of his patient, which would tend to prove that he had already attained a degree of fame and standing as a dentist. On the other hand, he informs us that he had been destined from his youth to surgery and that, because of reverses of fortune, he had been obliged to serve on ships of the King's navy as student surgeon. In view of the necessities of the time Colbert,[1] when organizing his fleet, had provided it with a medical staff and the conditions of admission to this service was not stringent; it was therefore probably after his discharge from the navy, where he must have stayed about three years, that Fauchard was able to devote himself to the specialty to whose elevation he was destined to contribute so largely. From this fact also it is difficult to imagine that he may have been born at a period much distant from 1675.

Accident alone did not lead Fauchard to direct his intelligent activity to the study of diseases of the organs of the mouth; many facts observed during his sojourn on the sea must have incited him. At that period one among others produced considerable ravages among sailors. Long

---

[1] COLBERT, JEAN BAPTISTE, Minister of Marine, born 1651, died 1690.

sea voyages rendered them subject to scurvy which had frequently if not always a disastrous effect upon the gums and consequently upon the teeth.

It is therefore certain that on shipboard, and particularly on warships containing large crews, surgeons found themselves obliged to care for these organs oftener than they would ashore. For these reasons alone Fauchard must have conceived the idea of specializing in the treatment of the mouth and teeth. Furthermore, he had the fortune to be the pupil at sea of A. Poteleret, surgeon-major of the navy, who had much experience in diseases of the mouth. Says Fauchard in the preface of his book, "I owe him the first glimmerings of the knowledge I have acquired in the surgery I practice, and the progress made with this able man gave me the enthusiasm which later conducted me to more considerable important discoveries."

Thus the lack of funds obliged him perhaps to direct his life to the practice of dentistry, more accessible than general surgery, which required longer and more costly studies.

It is clear that, after leaving the poorly paid service of the navy of the state, Fauchard was, in 1696, as we have seen, settled and favorably known in Angers, a city whose importance as a university center was greater than it is today. At that time real dentists, or "dentateurs," as they were still called, were few. Fauchard was the first to call himself "surgeon-dentist." Extraction of the teeth, which constituted current practice, was usually left to the barber, who joined to it bleedings, application of leeches and the manipulation of the instrument dear to M. Purgon. Nevertheless, the manual dexterity, which was developed through the extirpation of the teeth by means of the primitive instruments of the time, created and set apart a more specialized set of operators who had gradually enlarged the field of their intervention, such as the filing of carious teeth, removal of tartar, and even of benign tumors of the gums, etc., all still very rudimentary! Prosthesis, almost nonexistent, consisted of binding to the remaining teeth, by means of waxed thread or gold wire, clumsy imitations of human teeth carved from blocks of bone or of ivory.

The practitioners of the new art, when not located in large cities like Paris, found it necessary to travel in the neighborhood of their centers. Fauchard must also have had recourse to these journeys, first to add to his income, then to become better known so as to satisfy the many calls of a growing and improving practice.

Our information about his life and his wanderings at that period would have been limited if Fauchard had practiced his profession, like so many others, casually day by day and if he had not been gifted with the power of observation. He was not only an excellent observer, but he took joy in establishing the relationships between facts, hence he had the habit of taking notes of the cases or operations which struck him for their variety or their difficulty.

In his book, he described many of these cases all antedating the first edition. As it was he added very little to the text of this first edition either because his too large practice did not leave him time to note his observations or because of his age, for in 1728 the date of his first edition, he was nearing the fifties.

Thanks to his case-records and the activity he manifested at this period of his life we know that Fauchard, though established in Angers, ere long made trips to the towns of Anjou and later the adjoining provinces. We hear of him in Nantes, Rennes, Tours, etc., which he must frequently have visited until 1719. It is quite possible as some of his remarks may lead us to believe, that he made several trips to Paris. Perhaps he was summoned there by patients from the provinces who sometimes lived there, perhaps he went to study the ground with a view to establishing himself later.

We suppose, therefore, that Fauchard had sojourned in Paris. At any rate, until 1718 his headquarters were in Angers, where people of quality came from afar to consult him, as evidenced by the notes on a lady named Maubreuil, who came from Nantes to Angers to be treated by him.

His reputation in the whole of Western France was at its height, but he may have had the legitimate ambition to practice in a larger field—Paris, which had become more than ever the intellectual center of France and of Europe.

The increase of the population of Paris, which was considerable at that time, created in the large city needs which were profitable specially for those practicing the liberal professions. Dentists were naturally among the number, for the knowledge of the hygiene of the mouth was spreading and with it the call for more constant and more special treatment. There were then a number of practitioners enjoying some fame in Paris, such as a certain Carmeline, who mentions Fauchard with much praise.

At this time doubtless at the request of the "dentateurs" and perhaps of the Surgical College, a regulation was published in Paris, in 1700, stipulating that none could thenceforth practice as dentists without having a certificate as "expert," awarded after an examination before a commission of three Masters in Surgery appointed by the municipality.

This title did not always carry a guarantee of professional proficiency, as, remarks Fauchard in his preface, they had *naturally* omitted to add a specialist to the examining commission. As, meanwhile, there existed no textbooks where the prospective dentist might acquire the theory of his art, one is at a loss to determine what constituted the subject-matter of this examination, which was nevertheless demanded for a definite object.

In these favorable circumstances Fauchard already celebrated, came to settle finally in Paris, about 1718, for he notes in this his records that as early as 1719 Antonie de Jussieu had him summoned to operate upon a patient who had an enormous swelling caused by two carious teeth. Antonie de Jussieu, first of the illustrious dynasty, was a doctor of the Faculty of Medcine, member of the Academy of Sciences, Professor of Botany of the King's Garden, where he succeeded Tournefort. For such an important personage to have called on Fauchard the latter's reputation must already have been achieved in Paris or must have preceded him. Now in 1719 Fauchard must have been almost forty, had had twenty years of practice, and could have become sufficiently known—at a time when good dentists were rare—for the notabilities of science and medicine to have thought of having recourse to his skill and his guidance.

Established in Paris, Rue de la Comédie Française, directly in the University circle, Fauchard had almost at once a large practice. The best known physicians and surgeons, Dodart, State Counsellor, first physician to the King, to whom he dedicated his book, J. Louis Petit, Helvétius, de Jussieu, La Peyronie, Finot, Hecquet, Winslow and others, consulted him or referred patients. In 1725 the College of Surgery had even called him in consultation. It was with regard to a patient from Auxerre who was afflicted with an extensive and ulcerative tumor of the gums; the local surgeon had not dared to operate and had referred him to a colleague in Paris who, in view of the gravity of the case, proposed to have the advice of his colleagues united in a public session of the Surgical College of St. Côme. Fauchard was invited. After having given his opinion, he asked that the treatment be confided to him; he operated upon the tumor at his office with the assistance of several surgeons, treated the patient and cured him in three weeks.

All these occupations did not prevent him from thinking of his book and increasing the number of his observations. This work, which professionally speaking is so brilliant, supplies us such meager information on the private life of the author as to permit us only conjectures. I have endeavored to complete, through minute research in our national archives, what we can draw from Fauchard's book!

It would have been very important to discover the place of his birth. I had an exhaustive search made of the notarial records of the period which might perhaps have furnished information upon the principal features of the civil life and the financial status of our author; I have found nothing—nor anything in the archives of the parishes wherein he dwelt; besides, these archives were in large part destroyed, some at the time of the Revolution, some in 1871.

We have tried to deduce data about his character and his manner of thought with the assistance of his book and such portraits as we possess; but unfortunately they are insufficient. Fauchard had without doubt an amiable and courteous character. He was always reserved in the discussion of matters concerning his art. His numerous relationships in the medical world, a world quite disdainful and critical of those who did not belong to the confraternity of St. Côme, are certain proof of his amenity.

A few details now about the portraits which are essential factors of our biographical sketch.

The portrait which adorns his book was engraved by Scotin, after a painting by Le Bel. I have found no trace of this painting. Scotin, who engraved a number of works by Watteau, was not fortunate in his portrait of Fauchard, which lacks delicacy and character. I had the rare good fortune to discover and acquire another portrait in oils which is in my opinion a real work of art. This discovery has been one of the great joys of my life, so will you permit me to tell you how I became the possessor of this precious object? This portrait was part of the collection of Dr.

Cusco, surgeon in the hospitals of Paris, member of the Academy of Medicine, held in repute not only as an able and learned physician but also as a lover of art and of books, a musician, even a composer. He had brought together a large number of portraits of physicians and surgeons of the seventeenth and eighteenth centuries. I used to attend Dr. Cusco and about 1890 had the opportunity to see his paintings. I must admit that I had not particularly noticed Fauchard's portrait, and I believe Dr. Cusco did not suspect how deeply this picture would interest our profession, or he would have drawn my attention to it.

After his death, a sale was held at the Hotel Drouot and I went to it, knowing what rare treasures this collection contained. In trying to discover the names of the personages represented and admiring particularly this little portrait, I discovered on the back of the canvas the following inscription in ink—"*Pierre Fauchard, 1720*"—and further up on the frame, the name "*Netscher,*" presumably that of the painter.

In many encyclopedias Fauchard is simply described as a surgeon born in Brittany in the seventeenth century and dying in Paris in 1759; it is therefore not surprising that he should have figured as such in a collection of portraits of surgeons and physicians.

In this portrait Fauchard appears younger than in the book, but in both portraits he is dressed in about the same manner and has a similar pose.

Later investigations have convinced me that this was not painted by Netscher, I would prefer to attribute it to Rigaud or to one of his school; it may even possibly have been the work of F. Octavien, a painter admitted to the Royal Academy in 1725, with whom Fauchard was acquainted; one of his notes indicates that he had treated him. Continuing the investigation I found a few years ago a charming engraved portrait intended, as indicated by its size, for use as a frontispiece. This engraving, 9½ in. by 7½ in. represents the bust of a young man of thoughtful countenance, leaning on a table upon which is an *inferior maxilla;* in his right hand he holds an open book. The physiognomy recalls strikingly, but more youthfully, the portrait in oils which we have mentioned. I found this engraving without margins, without the name of the subject or of the engraver; it seems to be a proof, perhaps imperfectly finished, certainly a proof before the lettering.

However that may be, the presence of an inferior maxilla carries a sufficiently emblematic significance for us to reasonably assume that it represents a dentist. A surgeon or an anatomist, to whom it might apply, would probably not have been satisfied with so modest an anatomical emblem; a skull for example, would have had a nobler and less special meaning, at any rate more in harmony with the pompous and allegorical tone of the period.

If to these abstract deductions we join the concrete fact of the resemblance of this countenance to the portraits of Fauchard which we possess, we have a series of presumptions upon which to advance the statement that this portrait may well be the one which our author had engraved about 1723, when his manuscript was ready for the printer. If because of the five years' delay in its publication this engraving was not used, one may suppose that Fauchard became a celebrated practitioner by 1728, and disdained this presentment as no longer corresponding to his social and professional standing. In deference no doubt to the tastes of the day, he preferred the unskilful but theatrical plate of Scotin to this charming product of a freer graver, an abler design of a true artist.

To sum up, the portraits of Fauchard disclose him as graceful, a long thin nose slightly arched, shapely lips and mouth, expressing delicacy and kindliness; the eyes are large, pensive, with a look full of frankness. He bears upon his whole person the impress of that high French courtesy never more refined than at that time. The entire effect is one of a thoughtful, lovable and loyal man.

The Rue de la Comédie Française, which was also called Rue des Fossés, St. Germain, on the map of Paris made in 1734 after the plans of Turgot, later became the Rue de l'Ancienne Comédie. Despite all our search there is nothing left to identify for us the house Fauchard says he occupied upon his arrival in Paris in 1719. As for the Rue des Cordeliers, where he went to live in 1747, it is now named Rue de l'Ecole de Médecine; only thirteen houses are left, of which the last, number 13, is at the corner of the Place de l'Ecole de Médecine. Most of these houses have been rebuilt; the only old one, No. 5, has a large gate in the style of the eighteenth century; in the courtyard there still remains a domed building, which is the former amphitheater of the Royal College of Surgery and is at present the home of the School of Decorative Arts. Here again nothing remains to indicate the dwelling of Fauchard.

On the Place de l'Ecole de Médecine we see the large Gothic building reminding one of a church, which is now the Musée Dupuytren, and which is all that remains of the Convent of the Cordeliers, whose refectory it was; other parts of the convent were destroyed in 1790. Rue des Cordeliers, upon which stood the convent, was in the eighteenth century a little longer than the present Rue de l'Ecole de Médecine; it ran from the Rue de la Harpe to the Rue de l'Observance, near the Church of St. Côme and St. Damien.

We may assume that Fauchard's home was on the spot where the Boulevard St. Michel crosses, toward the corner of the Rue des Ecoles.

It was in 1747 that Fauchard having reached the age of seventy came to live, as he himself states in the second edition of his book, "in the street and near the Grands Cordeliers, Faubourg St. Germain, in a new house with a portecochère, where my sign will be on January 1, 1747."

The words "new house" would lead to the supposition that Fauchard had built this house; we shall show later that at the time he had accumulated a competence of respectable size.

Everything points to his having an active old age and that he still remained in active practice for a long while, no doubt to the great benefit of his sole pupil and successor, Duchemin.

He died in 1761, an octogenarian. The date of his death has been given as 1759, but no adequate proof is adduced. I am more inclined to believe that Fauchard died on the 22d of March, 1761, as stated by the celebrated anatomist and physician Portal, who published in 1770 a valuable "History of Anatomy and Surgery" in six volumes. We find on page 11, Vol. 5, a note on Fauchard and his work in which he gives for his death the date I have mentioned. Portal, a contemporary of Fauchard, was certainly acquainted with him as the eulogy in this book would permit us to believe. He is hence better qualified than others to settle this point, specially as his duty as a historian would constrain him to be as precise as possible. The cemetery of St. Côme and St. Damien was that of his parents and there he was no doubt buried.

The fame he had acquired outlived him and the title of pupil and successor to the celebrated dentist was claimed by several.

We are not certain that Duchemin succeeded him for long; we can only affirm that Duchemin was still in practice in 1759; we find proof of this in a treatise he published in that very year "On Caries of the Milk Teeth."

In any case Duchemin did not practice long after the death of his preceptor, for after we lose trace of the former a certain Delafondée, living "in the street and near the

Grands Cordeliers'' represents himself as the ''sole pupil and associate of M. Fauchard,'' as evidenced by the professional card which we possess.

The charming design which frames the card and bears the stamp of the delicate taste of the period is signed by Marillier, one of the greatest engraver designers of the eighteenth century.

The appearance of Fauchard's work was acclaimed as a professional event of the first magnitude and the first edition (1728) seems to have been rapidly exhausted. Five years later, in 1733, it was translated into German by Aug. Buddäus, royal counsellor and physician to the King, under the title ''Der Französische Zahnarzt,'' and was printed in Berlin. The portrait ornamenting this volume was engraved by J. B. Busch; it is nothing more than an imitation of the reversed proof of Scotin's. The unprecedented success of a book of this kind is revealed to us by the estimate placed upon it by Fauchard's contemporaries. We have first that of Landumiey, qualified practitioner, who was the dentists of His Catholic Majesty Philip V, King of Spain. Here is the text:

> I am too much interested in what may be advantageous to the public to fail to disclose by the present approbation, that I have never seen anything more perfect about all which concerns the teeth than the book which M. Fauchard has written. I find therein many thoughts and discoveries which are as sensible and useful as they are novel. The title of "Surgeon-dentist," which is at the head of this work, is well sustained through the knowledge collected by a brilliant mind, great observation and much assiduous labor. My experience in the author's profession leads me to extol with much pleasure the excellence of his treatise, which he gives with a disinterestedness as praiseworthy as it is rare.
>
> (Signed) LANDUMIEY.
>
> Paris, June 9, 1728.

Again, here is a testimonial which we find in a work published in 1746 by Bunon under the title ''Experiments and Demonstrations Made in the Hospital of Salpetriére and at St. Côme in the Presence of the Royal Academy of Surgery,'' by Bunon, surgeon-dentist of Paris.

Bunon, who specializes in the care of children's teeth, avers particularly how the teeth are affected by "rickets, scarlet fever, smallpox, phthisis and languor." He completely separated the process of dental erosion from the causes of dental caries. He was also a sagacious observer, whose opinion in the present case is very valuable to us. Paragraph 3 of the preliminary discourse in Bunon's book is devoted entirely to Fauchard. It is too long to be quoted here. Briefly summarized, he makes a very studied and fair analysis of Fauchard's books and declares his conviction that the author was a great master. Other surgeon-dentists, Leclause in 1754, Bourdet in 1768, etc., loudly sing his praises.

A no less precious testimony is furnished us by James Gardette who, with our other compatriot, Joseph Lemaire, introduced French dental art into the United States. Both followed Lafayette and Rochambeau to America in the capacity of naval surgeons in 1778. After the War of Independence, Gardette, who in Paris had been the pupil of the celebrated dentist, Le Roy de la Faudignére, became permanently established in Philadelphia in 1784.

From this city he wrote on March 30, 1791, the following curious letter to his brother, the engraver, living in Agen:

> Let me invite you, my dear Spiritou, to come to America, where I will make a dentist of you and make your fortune. I undertake to teach you and within a year to put you in a position to earn as much in an hour as you do in a week scratching copper in France. Without flattering myself my name will always enable you to do a good business in this country which is one of the best in the world. Come, my dear Spiritou, and count upon me as you should on yourself. If you decide to come and join me, you should be provided with some books upon my profession, and I would recommend:
>
> "The Surgeon-dentist," by M. Fauchard, two volumes.
> "The Art of the Dentist," by M. Bourdet, two volumes.
> "Diseases of the Teeth," by M. Bunon, two volumes.

As we see, in the opinion of Gardette who is considered in the United States more than Lemaire as the real introducer of the system of French dentistry, Fauchard's book, forty years after the author's death, was still the textbook recommended above all others to those who would practice our profession.

Even though Fauchard's book had not been translated, our colleagues in the United States profited none the less from his teachings, transmitted directly by Gardette. Hence the dentists of the United States, with the aptitude and practical sense they apply to all things, were enabled to reach, even as far back as the middle of the nineteenth century, the high reputation which they so justly enjoy.

As a consequence of the great political and social upheaval at the end of the eighteenth century, the name of Fauchard is almost entirely forgotten, a totally inexplicable oblivion. In 1835 the "Grand Dictionnaire de Médecine," in the articles on teeth, dentition, disease of the teeth, etc., treated at length with extensive bibliographical notes, fails to mention Fauchard's name anywhere except in the article on dental techniques, and yet quotes his contemporaries, Bunon, Jourdain, Lecluse, etc. What are we to think of J. E. Oudet, editor of these articles, who, although a practicing dentist, names Fauchard as a prosthetist and makes no mention of him as a surgeon-dentist?

Nevertheless, we must note that in 1821 Audibran, a highly endowed veteran of the profession, who, considering his age belonged to the host for whom the didactic value of Fauchard's book remained untarnished, says:

> The writings of Fauchard are still in our day the best in existence upon dentistry; these writings have brought forth the finest practitioners. It were impossible to describe more clearly or demonstrate more convincingly the precepts of an art which participates of both medicine and surgery; in this respect, whatever progress may have been made since, no work will bear comparison with the treatise of Fauchard.

Again, a few years later, in 1845, Trousseau, in his "Complete New Elements of the Science of Dentistry," praises Fauchard lavishly, saying he was "the Father and Founder of Dental Surgery."

Let us not forget that in 1863, Trousseau, one of the leaders in medicine, thought it worth while to publish in the medical journal *l'Abeille* a biography of Fauchard which, notwithstanding its importance, has unfortunately passed unnoticed by the larger number of our colleagues.

From 1880 on Fauchard's fame finally emerged from obscurity, as that year saw the dawn of a new glory for the memory of our great man. On the 13th of November, 1880, at the inauguration of the Dental School of Paris, my lamented friend, Dr. Louis Thomas, expressed himself thus in the course of an address upon the history of Odontology: "I have spoken of Pierre Fauchard; remember well that name, gentlemen, for with him opens a new era in the history of your profession."

Finally, may I state, not without pride, that a copy of Fauchard's portrait which I had sent, together with a biographical study, to the International Dental Congress of St. Louis, in 1904, was the occasion of a veritable apotheosis to the memory of Fauchard.

This offering elicited in the meeting a large enthusiasm and furnished an opportunity to the most eminent practitioners of the United States, among them Dr. Burkhart, Dr. Brophy, Dr. Grieves, Dr. Thompson, Dr. Trueman, Dr. Kirk and Dr. Thorpe to express their admiration for Fauchard's work, proclaiming him the founder of modern dentistry.

My colleagues having requested me to speak before you of Fauchard to celebrate his bicentenary, I have given you the result of my long study compiled in a manuscript wherein I analyze his work, and which I should have published ten years ago, only I still hoped to complete my work by the discovery of new documents. Then came the great war, which was the cause of new delay. I had assumed that nothing more would be found, when an extraordinary accident put me in touch last week with a lineal descendant of Pierre Fauchard—his great-grandson, M.

Robert Flüry-Herard, and for this I have to thank my young friend and colleague Bruschera. At last I had found the vein which I had fruitlessly sought for twenty-five years! Today, then, all is clear, we have access to papers, deeds, official documents, etc., which the family possesses and in them we find definite information with regard to Fauchard's family life.

I can not resist the pleasure of giving you the high lights of what I have just learned. And first, it is most fortunate for my research and deductions that they turn out to be in accord with the truth. I had, as you remember, placed the date of his birth as between 1675 and 1680; the documents reveal it as being in 1678. If the family knew very little of the youth and of the first period of Fauchard's professional life they were aware that he was a celebrated practitioner of the eighteenth century, but the account which I have given you was almost unknown to them. They knew also that at the time of his marriage in 1729 he was quite wealthy and that in 1734 he had purchased at auction a vast estate, with the château called "de Grand Mesnil," near Orsay, at the entrance to the Chevrence Valley.

This château, built in 1629 by the original owner, Antonie des Valles, is a pure specimen of Louis XIII style, the windows outlined in brick, the high French roof recalling the most elegant buildings of the period. This château remained the property of Fauchard's descendants until 1920, that is to say almost two centuries. The portrait by Le Bel to which we have referred is yet in the family, as well as one of Mme. Fauchard.

Pierre Fauchard married in 1729 Mdlle. Elisabeth Guillemette Chemin, daughter of Pierre Chemin, former royal counsellor, notary syndic and keeper of the seal of the community of royal and apostolic notaries of the city and seneschal court of Rennes. The marriage contract is dated August 17, 1729, and therein Fauchard is designated as a surgeon-dentist, residing in Paris, Rue des Fossés, St. Germain, parish of St. Sulpice.

He had an only son born in 1737, Jean Baptiste Fauchard, who was an advocate in Parliament, then counsellor to the Admiralty of France. Later, having opposed the Parliament of Maupeou, he had to leave France, and possessing real talent as an amateur comedian, took an engagement at the theater of Brussels. On his return from exile in 1790, he made his debut at the Comédie Française. A pensioner in 1791, Jean Baptiste de Grandmesnil (the name of his domain which he had adopted for the stage) was received into the society of the theater and became a celebrated comedian, friend and contemporary of Talma, who, we may mention in passing, was a dentist himself, also the son of a dentist. Grandmesnil achieved his greatest success in the comedies of Moliére, while Talma was more renowned in tragedy.

In the foyer of the Comédie Française may be seen a portrait of Grandmesnil in the rôle of *l'Avare.*

When the ordinance was passed establishing a class in elocution at the conservatory, de Grandmesnil, celebrated and distinguished, was named professor, the first occupant of the new chair. He completed his glorious career as a member of the Institute of France. He died in 1816.

The life of de Grandmesnil is naturally far better known that that of his father, and he is regarded by the family as its great man.

I can not enlarge further upon this today, but I intend to put these documents in order and to make them known.

Let us be thankful to chance which so often grants favors, but also to M. Robert Flüry-Hérard, who has been so extremely obliging as to place at my disposal this documentary treasure which will permit us to become acquainted with Pierre Fauchard's life in its minutest details.

We are happy to greet in our midst today Mme. Flüry-Hérard and her son.

Chance, once more, smiles upon us at this time. M. Dagen, who under the pen-name of Montcorbier, contributes to the magazine *La Semaine Dentaire* historical sketches of dentistry, has just published, without my previous knowledge, an interesting work upon Fauchard. We find there a document of the greatest importance—the death certificate of Pierre Fauchard for which as I have said I had sought in vain. Here is this certificate, which supplies some supplementary information and specially the place of his interment: The Church of St. Côme and St. Damien, which was in the Rue des Cordeliers, near the spot where the Rue de l'Ecole de Médecine crosses the Boulevard St. Germain.

*Extract from the Register of Baptisms, Marriages and Interments of the Parish of St. Côme and St. Damien, in Paris.*

On March 23, 1761, was buried in the nave of St. [redacted]ement, the body of Pierre Fauchard, former master surgeon-dentist, Seigneur du Grand Ménil, widower in second marriage of Elisabeth Chemin and husband of Louise Rousselot, deceased the day before yesterday, aged 83, Rue des Cordeliers, this parish. Were present: Jean Baptiste Fauchard, Seigneur du Grand Ménil, his son, advocate to the Parliament; Jean Baptiste Béllissen, advocate to the Parliament and commander registrar, general secretary of the Hospitaler Order of St.-Esprit, both rue and parish St.-André-des-Arcs, who have signed with us.

J. B. FAUCHARD DU GRAND MESNIL,

DE LA ROUE. DE BELLISSEN.

(Archives of the Seine, Reconstitution des actes de l'état civil, 12 Août 1876, No. 437242 X.)

Compared with the original by myself, vicar priest, keeper of the register of the said parish.

GIRARD.

Notarial oath received July 9, 1762, by Me. Robineau.

There is every reason to warmly thank M. Dagen for the discovery of this document.

Here, in addition, is the notice of his funeral which has been forwarded to us by M. d'Aubigné, genealogist of the Bibliothéque Nationale.

M.

You are requested to attend the procession, service and interment of Monsieur Pierre Fauchard, former master surgeon-dentist, Seigneur de Grand Ménil, deceased at his residence, Rue de Cordeliers, which will take place Monday March 23, 1761, at ten o'clock in the morning at the Church of St. Côme, his parish, where he will be buried.

*Requiescat in Pace.*

His widow, Madame Fauchard, and his son, Monsieur Fauchard de Grand Ménil, advocate to the Parliament.

The Church of St. Côme was demolished in 1836, but the remains of Pierre Fauchard were not transferred to the family vault at the château of Grandmesnil, where lies his son.

It appears from this barren attempt at reconstruction that we do not yet know the place of his birth nor the exact location of his burial. Nevertheless, by a striking coincidence, we are gathered today in this Sorbonne, situated only a few paces from the house wherein he dwelt, where he died, and from the presumed spot of his sepulcher.

Fauchard having been born in 1678, his bicentenary might have been celebrated in 1878, but he says he completed his manuscript in 1723, that is to say exactly two hundred years ago; it is therefore this remarkable work which we celebrate today. But it is not only to celebrate his bicentenary that we are gathered here, we also unveil his statue. I must voice the rare good fortune of having for a friend the talented sculptor of this bust, Paul Paulin, whose works we have all admired in our museums and at the Faculté de Médecine. He has followed my researches and said it was possible to make a composite of the portraits to model the bust.

As a sculptor and an odontologist was he not particularly qualified to reproduce the features of Pierre Fauchard?

It is a happy occasion for us, my dear Paulin, to convey to you our admiration and to say to you how proud we are that one of our number should have become the great sculptor which you are.

Our French colleagues, those of the United States and of all countries, unanimously applaud the magnificent work which you have bestowed upon our profession, and in their name I express to you their profound gratitude.

